# Cretinism and the Puerperium: Sequel to a Recorded History
^1^Read at a meeting of the Bristol Medico-Chirurgical Society, April 14th.


**Published:** 1909-06

**Authors:** David A. Alexander


### CRETINISM AND THE PUERPERIUM: SEQUEL TO A RECORDED HISTORY.[1]

BY

DAVID A. ALEXANDER, M.B.

I SAW Mrs. M. H. for the first time at the end of October, 1908, when she was in the seventh month of her fourth pregnancy, and expecting her confinement in January.

She was twenty-six, of slight physique, rather sallow complexion, cheerful and intelligent expression. Inquiry into her present and past history elicited the fact that she had been accustomed to take thyroid extract.

She gave the following account: She had recently come to Bristol, having been born and brought up in Edinburgh. She had good health and was counted a quick child until the age of ten. Then she stopped growing and became dull, and her friends, concerned on her behalf, took her during the next four years from doctor to doctor; and although by one at least, she understood, the nature of her complaint was recognised, treatment was not efficiently carried out. While casually attending the out-patient department of the Edinburgh Royal Infirmary, she attracted the notice of Dr. Byrom Bramwell. Admitted an in-patient, thyroid extract was adequately administered, and she began to recover energy, intelligence and growth.

After her dismissal its use, in frequent courses, was continued, and with success. She quickly passed from childhood to adolescence, and at the age of twenty she entered the married

1 Read at a meeting of the Bristol Medico-Chirurgical Society, April 14th.

state. It had been her experience that her need of the extract had continually lessened; dryness of the skin was the first of the symptoms to guide her. In her three previous pregnancies she had taken it, but not more frequently than at other times, and since the beginning of the present pregnancy she had required none. Of her three children the first and the third were alive and healthy, the second had suffered from a wasting disease, and had died of "meningitis." Further examination disclosed no signs of myxœdema, although the thyroid gland could not be felt; neither were there any of the symptoms of renal toxæmia associated of late with thyroid insufficiency. The urine, when examined, proved to be normal. Sickness in the early months of gestation had not been excessive, and everything pointed to a normal completion at term.

Summoned to see her at the end of December, in the beginning of the ninth month of her pregnancy, I found her in circumstances of some discomfort, as she had been changing her lodgings. There had been a profuse uterine hemorrhage, the cause of which could not be determined; the placenta was not involved in the os; labour was in progress, the head presenting, and with little delay a male child was born, without mechanical interference or perineal laceration.

During the first week of the puerperium the patient's condition was all but wholly satisfactory—lochia scarcely offensive, a temperature oscillating one degree, the skin somewhat dry, occasional fugitive pains, something of weariness and apathy—slight as those symptoms were, they gave an emphasis to the patient's desire to resume thyroid extract, the eliminant action of which upon skin and kidneys could not but be advantageous.

On the eighth night delirium was reported; on the ninth evening occurred a rigor, with temperature of 105, and pulse rate upwards of 130. The condition on the tenth day was reassuring; on the following the skin became icteric; on the twelfth the jaundice was intensified, and accompanied by numerous petechiæ. The typhoid state supervened. She was now admitted into Cossham Memorial Hospital, under the care of Dr. Bertram Rogers, but not before the appearance of venous thrombosis of the left leg, (purulent) synovitis of the left knee, and subcuticular abscesses in pressure areas, attested a puerperal pyæmia. She died on the fourteenth day.

No autopsy was obtainable, so that it was impossible to ascertain the exact state of the thyroidal system, or its relation to recent morbid changes. The latter had probably been of the usual type. If the early symptoms, including the hemorrhage in parturition, had actually been expressive of athyroidea, there was, perhaps, room for taking the icterus to be that of one of the graver forms of the liver derangements of pregnancy. There had been, however, no diminution in liver dulness, and the scanty urine yielded neither leucin nor tyrosin. Of the occurrence of yellow atrophy in the puerperium, examples [7] are not wanting to supply an exception to Rokitansky's observation that "the severe jaundice affecting women during the puerperal state is always dependent upon pyæmia, and never upon an appreciable derangement of the liver."

Dr. Byrom Bramwell, with whom I communicated, kindly points out the earlier record of the patient in his work, *Anæmia, and Diseases of the Blood-Forming Organs and Ductless Glands*[1] (p. 371). He describes the case as one of juvenile myxœdema, using the term in contradistinction to cretinism, in which the greater defect and deformity are due to the same morbid process, beginning in infancy. The family history included phthisis, insanity and convulsions. The patient had measles in childhood, herpes zoster in her twelfth year (after the onset of myxœdema). When she stopped growing, at ten, permanent dentition was backward. Myxœdema was already developed before intelligence flagged. As to the features, the nose and nostrils were flat and wide, and these features persisted in later life, but without the grossness of cretinism; and in this type her children resembled her. The hair was scanty; after recovery it became luxuriant, and so remained, a proof of good integumentary nutrition.

The menstrual cycle, once established, had since remained normal. The patient responded to treatment in some six weeks, and the extract then administered was what she had since taken, never exceeding five grains three times daily.

No thyroid gland could at any time be felt. But one cannot think that it was absent during her early years of growth, or that if it had been wholly lost by a destructive lesion she could afterwards have done without her supplies for long periods together. But a gland, always small and feeble, may have exhibited[2] temporary and relative insufficiency, and, with narrow limits of activity, may have required frequent reinforcement from without.

The recent application of thyroid extract to the toxic disorders of pregnancy tends to foster the impression that child-bearing must necessarily aggravate athyrea. But exceptions have been observed, and several are cited in the Clinical Society's report upon myxœdema.[3] An explanation of the anomaly is offered by the theory of vicarious fœtal thyroidisation, and I am indebted to Dr. Edgeworth for calling my attention to it in connection with the present case. Operating upon bitches, Halsted found that the thyroidal system could be almost wholly extirpated in the pregnant animal without ill-effects; and that in the puppies, born at term, the thyroid glands were enlarged. Edmunds[4] corroborated these experiments, and satisfied himself that the increase in the size of the gland in the young was compensatory, and presumably due to the functional activity imposed upon it by the maternal deficiency.

For the proof of such fœtal activity in any case one would look for enlargement in the gland of the child. In the present case it must be said that it was not apparent; there was nothing unusual in the infantile contours of the neck.

It would also seem to follow, in such cases, that with the loss of her supply, on the completion of pregnancy, a mother might be liable to acute symptoms of myxœdema.

As to the sequence of disease in mother and child, as observed in areas of endemic cretinism, it is to be remarked that both are simultaneously exposed to the same causative agent—an organism,[5] perhaps, which destroys the developing gland in the unborn child, or is transmitted to the infant in the course of lactation.[6] These consequences were not to be expected in this instance.

After-histories in the recovery of cretins, as yet, can be but few, and as a contribution to the subject these particulars are recorded.

*Discussion.*—Dr. Edgeworth asked if the infant, which had been born, had enlargement of the thyroid gland. It had been shown that bitches with half the gland removed had pups with large thyroids. Possibly the woman had been able to do without thyroid extract during pregnancy because the child had a large thyroid, the secretion from the child's thyroid being sufficient both for the mother and child.—Dr. Alexander replied that the infant's thyroid was not enlarged.

REFERENCES.

1 Bramwell, *Anæmia and Diseases of the Blood-Forming Organs and Ductless Glands*, 1899, p. 371.

2 Berry, *Diseases of Thyroid Gland*, 1901: chap., "Atrophy and Hypertrophy."

3 *Tr. Clin. Soc. Lond.*, Report on Myxœdema, 1888, xxi, sheet 8.

4 Edmunds, Erasmus Wilson Lecture, 1901, *Lancet*, 1901, i, 1317 *et seq.*

5 McCarrison, "Cretinism in Gilgit," *Lancet*, 1908, II, 1275 (*cf.* Seligmann, *J. Path. and Bacteriol.*, 1904, ix, 311.)

6 Tedeschi, *Lancet*, 1908, i, 1310, "Cretinism in Italy."

7 Sturmer, "Atrophy of Liver with Jaundice," *J. Obst. and Gynecol. Brit. Emp.*, 1903.

